# The Usefulness of Impedance Cardiography for Predicting Beneficial Effects of Cardiac Rehabilitation in Patients with Heart Failure

**DOI:** 10.1155/2013/595369

**Published:** 2013-08-26

**Authors:** Grzegorz Gielerak, Paweł Krzesiński, Ewa Piotrowicz, Ryszard Piotrowicz

**Affiliations:** ^1^Department of Cardiology and Internal Diseases, Military Institute of Medicine, Szaserow Street 128, 04-141 Warsaw, Poland; ^2^Telecardiology Center, Institute of Cardiology, Alpejska Street 42, 04-628 Warsaw, Poland; ^3^Department of Cardiac Rehabilitation and Noninvasive Electrocardiology, Institute of Cardiology, Alpejska Street 42, 04-628 Warsaw, Poland

## Abstract

*Aim*. Cardiac rehabilitation (CR) is an important part of heart failure (HF) treatment. The aim of this paper was to evaluate if thoracic fluid content (TFC) measured by impedance cardiography (ICG) is a useful parameter for predicting the outcome of CR. *Methods*. Fifty HF patients underwent clinical and noninvasive haemodynamic (TFC) assessments before and after 8-week CR. *Results*. As a result of CR, the patients' exercise tolerance improved, especially in terms of peak VO_2_ (18.7 versus 20.8 mL × kg^−1^ × min^−1^; *P* = 0.025). TFC was found to identify patients with significantly improved peak VO_2_ after CR. “High TFC” patients (TFC > 27.0 kOhm^−1^), compared to those of “low TFC” (TFC < 27.0 kOhm^−1^), were found to have more pronounced increase in peak VO_2_ (1.3 versus 3.1 mL × kg^−1^ × min^−1^; *P* = 0.011) and decrease in TFC (4.0 versus 0.7 kOhm^−1^; *P* < 0.00001). On the other hand, the patients with improved peak VO_2_ (*n* = 32) differed from those with no peak VO_2_ improvement in terms of higher baseline TFC values (28.4 versus 25.3 kOhm^−1^; *P* = 0.039) and its significant decrease after CR (2.7 versus 0.2 kOhm^−1^; *P* = 0.012). *Conclusions*. TFC can be a useful parameter for predicting beneficial effects of CR worth including in the process of patients' qualification for CR.

## 1. Introduction

Cardiac rehabilitation (CR) as an element of secondary prevention of heart failure (HF) results in a number of clinical benefits. It reduces all-cause mortality, morbidity, and the incidence of adverse cardiovascular events and improves the quality of life. However, not all HF patients benefit from monitored exercise training (ET), and the reasons for this have not been unequivocally identified [1–4]. Patients who start CR are characterised by various clinical presentations, especially in terms of haemodynamic status, exercise tolerance, and treatment optimisation.

The mechanisms underlying the adaptation to physical exercise are known to deteriorate in the course of HF, which may significantly limit the benefits of CR. On the other hand, there is a group of optimally managed patients with a relatively good exercise tolerance who do not derive any additional benefit from CR. In the light of the limited access to supervised CR, it seems justified to search for a tool to identify those HF patients who have the greatest chance to benefit from CR.

In a previous study, we demonstrated the usefulness of impedance cardiography (ICG) in the evaluation of the effect of CR [5]. This simple method of noninvasive cardiac monitoring enables the estimation of numerous haemodynamic parameters, such as cardiac output (CO) and thoracic fluid content (TFC), and is increasingly employed in the diagnosis and treatment of HF [6–8]. The assessment of TFC has also proved to be of value in predicting the effectiveness of defibrillation during the testing of implantable cardioverter-defibrillators [9]. It cannot therefore be ruled out that TFC might have a prognostic value in case of other therapeutic interventions.

The assessment of peak VO_2_ with cardiopulmonary exercise treadmill test (CPET) is the reliable method of estimation of exercise capability, especially in patients with HF [10] and its change after treatment can have important clinical implications. It was observed that improvement in peak VO_2_ is associated with lower mortality. On the other hand, a lack of beneficial effect in peak VO_2_ after CR is the independent predictor of adverse cardiovascular events in the future [11–13].

The aim of the retrospective analysis presented in this paper was to evaluate the hypothesis that TFC is a useful parameter for predicting the outcome of CR.

## 2. Methods

### 2.1. Study Population and Protocol

We studied 50 patients (44 men; mean age 56.2 ± 8.8 years) with HF in stable clinical condition qualified for CR in the Department of Cardiac Rehabilitation and Noninvasive Electrocardiology (Institute of Cardiology, Warsaw, Poland). To assure relatively homogenous study group, inclusion criteria were defined as (1) systolic HF regardless of its aetiology, defined according to the European Society of Cardiology (ESC) guidelines [10] and diagnosed at least 3 months before study enrollment; (2) LV ejection fraction (LVEF) ≤40% as assessed by echocardiography; (3) New York Heart Association (NYHA) class II-III; and (4) stable clinical condition and optimal treatment that was not modified during the last 4 weeks before study enrollment.

All of the patients underwent detailed screening before recruitment to exclude any diseases that could strongly influence exercise capability, training compliance, and haemodynamic status. Exclusion criteria included (1) NYHA class I or IV; (2) unstable angina; (3) an acute coronary syndrome within the last 4 weeks, coronary artery bypass grafting within the last 8 weeks, or initiation of cardiac resynchronization therapy (CRT) within the last year; (4) symptomatic or exercise-induced arrhythmia or conduction disturbances; (5) valvular heart disease or other acquired cardiac conditions requiring surgical intervention; (6) hypertrophic cardiomyopathy; (7) severe pulmonary hypertension or other severe lung diseases; (8) uncontrolled hypertension; (9) anemia (haemoglobin level < 10.0 g/dL); (10) acute and/or decompensated noncardiac disease; (11) impaired motor function due to severe musculoskeletal or neurological disease; (12) severe or chronic inflammatory disorders; (13) neoplasm; (14) severe mental disorder; and (15) lack of patient consent to participate in the study.

Drug therapy used prior to initiation of CR was not modified during the training. Demographic and clinical characteristics of the study group are shown in Table 1.

During this prospective study, all patients underwent clinical assessment before and after 8 weeks of CR that included clinical examination with evaluation of symptoms using the NYHA classification, echocardiography, six-minute walk test (6-MWT), cardiopulmonary exercise treadmill test (CPET), and ICG. The study was approved by the local ethics committee at the National Institute of Cardiology, and all patients gave written informed consent for the participation in the study.

### 2.2. Echocardiography

Two-dimensional echocardiography was performed using standard parasternal, apical, and subcostal views (VIVID 4 GE Medical System, 2.5 MHz transducer). The left ventricular ejection (LVEF (%)) was analyzed using the biplane Simpson technique.

### 2.3. Cardiopulmonary Exercise Treadmill Test

Cardiopulmonary exercise treadmill test (CPET) was done at the same time in the morning for all patients, approximately 2 h after their morning medications and a light breakfast. Each subject performed a symptom limited CPET according to a ramp protocol, as recommended by the American Association of Cardiovascular and Pulmonary Rehabilitation [14]. The test was performed using a Schiller treadmill (Carrollton, USA) which was connected to a computerized breath-by-breath spiroergometry system (ZAN 600, ZAN Messgeräte GmbH, Germany). Oxygen consumption (VO_2_) was measured continuously using breath-by-breath analysis and used as an index of exercise capacity. Peak VO_2_ (mL × kg^−1^ × min^−1^) was defined as the highest oxygen uptake level achieved during the final 30 s of CPET. The formula used for the prediction of VO_2_ (peak VO_2_% N) was the Wasserman standard calculation, which incorporates sex, age, height, and weight of the subject and is valid for patients aged over 20 years [15]. A 12-lead electrocardiogram (ECG) and heart rate (HR) were recorded continuously at rest, during the CPET, and during recovery until HR, ECG, and VO_2_ returned to the baseline values. Blood pressure was measured manually every 2 min using a sphygmomanometer. Subjects were encouraged to exercise until they reached a self-determined limit of their functional capacity (perceived exertion or dyspnoea) or until the physician terminated the test according to the ESC guidelines [16]. None of the patients were limited by angina. The patient's subjective level of perceived exertion was quantified every minute during and at the end of CPET using the Borg (6–20) scale [17].

### 2.4. Six-Minute Walking Test

This was conducted using a standardized protocol between 11 AM and 2 PM after taking the usual medication [18]. Patients were required to perform a 6 min shuttle walk test with markers placed at 25 m. The distance in 6-MWT was analyzed.

### 2.5. Impedance Cardiography

All ICG measurements were performed using a Niccomo device (Medis, Germany) in a supine position and after 10 min of rest. Date were recorded during a 10 min study and exported to a dedicated software (Niccomo Software). In this study, we analyzed one of the measured parameters—TFC (its absolute values and change after CR).

### 2.6. Exercise Training

Exercise training (ET) was planned individually for each patient in line with the published guidelines [19–21]. The chosen workload reflected individual effort tolerance with regard to (1) perceived exertion according to the Borg scale and (2) the training HR range where the assumption was that patients should not exceed perceived moderate exertion during ET (i.e., a score of 11 on the Borg scale). The training HR was calculated using the method known as HR reserve. This method uses a percentage of the difference between the maximum HR and the resting HR and adds this value to the resting HR [22]. The target training HR was 40–70% of the HR reserve. Following baseline evaluation during the hospitalization, patients underwent a few, usually three–five, monitored educational ET sessions, during which HR at the perceived moderate exertion level was established. After that, all patients underwent an 8-week comprehensive home-based cardiac telerehabilitation. A training session consisted of three parts: (1) a warmup lasting 5–10 min, consisting of breathing, light resistance exercises, and calisthenics, (2) an aerobic endurance training based on walking training for 30 min, and (3) a 5 min cooling down period. Patients trained three times a week. The methodology of monitoring and education has been described previously [23–25].

### 2.7. Statistical Analysis

The statistical analysis of the results has been performed using Statistica 7.0 (StatSoft, Inc.). The distribution and normality of data were assessed by visual inspection and using the Shapiro-Wilk test. Continuous variables were presented as means ± standard deviations (SD) and categorical variables as absolute and relative frequencies (percentages). Assessments of CR outcomes and between-group comparisons were performed using the Student's *t*-test for normally distributed data and using nonparametric tests for data that did not show a normal distribution. Linear correlations were defined using Pearson's correlation coefficient. Absolute changes in the study parameters were calculated by subtracting the pre-CR value of a given parameter from its post-CR value. In the qualitative analysis, changes of more than 5% were considered significant. A *P* value of <0.05 was taken to indicate statistical significance.

## 3. Results 

### 3.1. The Outcomes of Cardiac Rehabilitation in the Study Population

CR resulted in increased exercise tolerance in terms of improved peak VO_2_, peak VO_2_% N, and the 6-MWT distance (Table 2). Furthermore, an improvement in the NYHA class was seen in more than 30% of the patients. ICG performed after the CR also showed a reduction in TFC. These results have been presented and discussed elsewhere [5].

### 3.2. The Outcomes of Cardiac Rehabilitation in TFC Subgroups

Median baseline TFC in the study population was 27.0 kOhm^−1^ (range: 17.5–37.7 kOhm^−1^). The patients were therefore divided into two subgroups: a subgroup with TFC values above 27.0 kOhm^−1^ (high TFC) and a subgroup with TFC values below 27.0 kOhm^−1^ (low TFC). A significantly greater improvement of peak VO_2_ and peak VO_2_% N and a significantly greater TFC reduction were observed in the “high TFC” subgroup. At the same time, these patients had lower BMI values. The prevalence of treatment with diuretics was significantly higher in the “low TFC” subgroup (Table 3). No significant differences were observed in the other parameters.

### 3.3. The Outcomes of Cardiac Rehabilitation in Subgroups Distinguished by Peak VO_2_ Improvement

**Figure 1:**
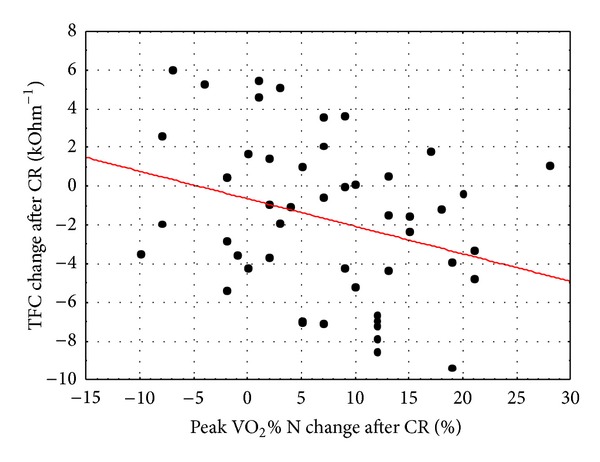
Correlation between change in TFC and peak VO_2_% N.

No clinically relevant increase in peak VO_2_ after CR was observed in 36% of the patients (with some of these patients showing a decrease). The patients in whom peak VO_2_ increased after CR were characterised by significantly higher baseline TFC values and a greater reduction of the value of this parameter following CR (Table 4). The change in TFC significantly correlated with the change in peak VO_2_% N (*r* = −0.304; *P* = 0.032)—Figure 1, peak VO_2_ (*r* = −0.305; *P* = 0.031), and LVEF (*r* = 0.307; *P* = 0.030). We observed no distinct interrelation between the beneficial change in peak VO_2_% and the other parameters.

## 4. Discussion

The quality of life in HF patients largely depends on ability to ET, which is why CR plays a special role in the management of HF. However, not all the patients show improvement in functional parameters after completion of a CR cycle. In the face of an epidemic of HF at the beginning of the 21st century, the search for diagnostic methods that would help identify patients who would benefit the most from CR seems particularly justified. ICG seems to be a useful tool for predicting the outcome of CR.

### 4.1. Predictability of the Effectiveness of Cardiac Rehabilitation

As is commonly known, not all the patients who undergo CR achieve an improvement in exercise capability. The variable clinical effect of CR is undoubtedly due to complex pathomechanism. Excessive sympathetic stimulation is reported to be one of the reasons for the lack of favourable response to CR. It has been demonstrated that a lower chronotropic reserve and a slower heart rate recovery (as two interrelated parameters) are signs of parasympathetic dysfunction and are associated with a poorer response to CR and an unfavourable prognosis [12].

Another factor that correlates with the outcome of CR and prognosis in patients undergoing ET is systolic blood pressure recovery, a parameter that characterises the rate at which systolic blood pressure returns to baseline after exercise. Sheikhvatan et al. [26] showed that baseline systolic blood pressure recovery rate allows to predict the success of CR. This study points out the fact that impaired normalisation of blood pressure after exercise may be accompanied by a poorer exercise capability and impaired control of the autonomic nervous system in terms of vascular resistance modulation [26]. A more detailed assessment of the left ventricular systolic function may also be useful for predicting the outcome of CR. For example, Smart et al. [27] observed that the change in peak VO_2_ as a result of CR correlated with the baseline value (*r* = 0.51; *P* = 0.003) and the change (*r* = 0.44; *P* = 0.01) in left ventricular strain assessed by tissue Doppler imaging.

It seems that the paradoxical finding that patients in a better clinical condition improve relatively less after ET is not accidental. It turns out that the greatest improvement in peak VO_2_ following CR is observed in patients with the greatest baseline impairment of exercise capability [13, 28, 29]. Rocha et al. [30] have also demonstrated that the greatest improvement of exercise capability is seen in older and less fit patients.

### 4.2. Impedance Cardiography

The factors affecting the outcome of CR and haemodynamic mechanisms underlying the clinical response to ET in patients with HF continue to be investigated in clinical trials. The baseline haemodynamic status should be, beyond any doubt, considered in the planning of ET and the course of CR.

On the base of previous studies [31–35], we considered ICG to be potentially useful for haemodynamic monitoring in patients undergoing CR. ICG enables the assessment of clinically relevant parameters, among which CO and TFC seem particularly important in the case of HF patients. In our study population, CR led to a reduction in TFC but only in the group of patients with relatively higher values of that parameter. What is most important is that this phenomenon was accompanied by a significant increase in peak VO_2_. In patients with low baseline TFC, cardiac rehabilitation did not lead to such marked improvement in exercise capability measured by peak VO_2_. Given the fact that physical exercise in HF patients decreases preload [36], it is reasonable to assume that the “high TFC” subgroup benefited as a result of this mechanism. Although they were clinically stable and with TFC values within a range (27.0–37.7 kOhm^−1^) that only slightly exceeded 35 kOhm^−1^ (the value proposed by Packer et al. [33] as a cutoff value for elevated risk of HF decompensation), ET effected a further reduction of TFC accompanied by an improvement in exercise capability. No such beneficial change in the haemodynamic profile was observed in the “low TFC” subgroup. The improvement of neurohormonal balance after CR—that was observed by other investigators—could be the important effect of ET explaining the lowering of TFC [37]. Lower prevalence of treatment with diuretic in the “high TFC” subgroup can be considered as an explanation of baseline “subclinical fluid overload.” However, these observations do not find clear confirmation in the analysis of subgroups distinguished by peak VO_2_ improvement, where clinical benefit was associated with more frequent use of diuretics.

In the context of quite surprising finding that the patients with a relatively low TFC benefit less from CR, it could be hypothesised that in HF patients, a nonlinear relationship between the clinical condition and the fluid status may exist (similar to the J-curve of blood pressure distribution). This hypothesis is also supported by the observation that excessively intensive diuretic use in HF patients adversely affects their clinical condition [38]. The relevant dependence of cardiac pump function on the Frank-Starling mechanism can play an important role in this phenomenon. In healthy individuals, left ventricular systolic function principally depends on the inotropic properties of cardiac myocytes, while in patients with advanced HF, the contractile response to stretching is the predominant component of the left ventricular contraction mechanisms [39–43]. The evaluation of TFC may therefore be important for predicting the outcome of CR.

In our study, we did not observe any effect of CR on LVEF. These results confirm the majority of observations that showed minimal or no change of LVEF after CR and poor correlation between peak VO_2_ and LVEF [19, 44, 45]. However, the lack of increase in resting LVEF does not exclude hemodynamic improvement in left ventricular function, especially during exercise. Although HF patients are predominantly dependent on systolic dysfunction, abnormal left ventricular filling dynamics and impaired relaxation also play an important role in low exercise performance [40]. ET can elicit increase in cardiac output by improvement in exercise maximal stroke volume because of the decrease in end-diastolic volumes and pressures, even with no parallel alternation in resting LVEF [19, 44]. As we suggested in our previous study [5], the clinical benefits of CR may be partly explained by an increase in preload-dependent contractile reserve and reduced LVEF dependency on the Frank-Starling mechanism.

### 4.3. Limitations

The authors are aware that the small sample size and the retrospective design are limitations of the study and that the evaluation of the outcome of CR directly after its completion may not be equivalent to long-term outcomes. It should be expected that the results obtained in a larger group of patients would be characterised by a higher statistical power. At the same time, haemodynamic monitoring of exercise using ICG, which is currently possible (using, for instance, the portable device PhysioFlow (Manatec Biomedical, France)), would be a valuable complement. We are also aware of some bias to males that stated 88% of the study population and lower BMI and dyslipidaemia prevalence in the “high TFC” subgroup.

## 5. Conclusions

Thoracic fluid content measured by impedance cardiography revealed to be a useful parameter in predicting beneficial effects of cardiac rehabilitation. Patients with higher thoracic fluid content seem to benefit more from cardiac rehabilitation when their fluid status improves. Thoracic fluid content may therefore be a parameter worth including in the process of patient qualification for cardiac rehabilitation.

## Figures and Tables

**Table 1:** Baseline characteristics.

	Study group (*n* = 50)
Men, *n* (%)	44 (88.0)
Age (years)	56.2 ± 8.8
LVEF (%)	30.0 ± 7.5
NYHA class II, *n* (%)	30 (60.0)
NYHA class III, *n* (%)	20 (40.0)
BMI (kg × m^−2^)	28.7 ± 3.8
HF aetiology, *n* (%)	
Ischaemic	42 (84.0)
Nonischaemic	8 (16.0)
Past medical history, *n* (%)	
Myocardial infarction	38 (76.0)
Diabetes	14 (28.0)
Dyslipidaemia	39 (78.0)
Hypertension	26 (52.0)
Medications, *n* (%)	
ACE inhibitor	46 (92.0)
Angiotensin receptor blocker	6 (12.0)
Beta-blocker	50 (100.0)
Loop diuretic	40 (80.0)
Spironolactone	47 (94.0)
Aspirin	43 (86.0)
Statin	46 (92.0)

ACE: angiotensin-converting enzyme, BMI: body mass index, CABG: coronary artery bypass grafting; HF: heart failure; LVEF: left ventricular ejection fraction; NYHA: New York Heart Association.

**Table 2:** Comparison of analysed parameters before and after CR (mean ± SD).

	Before CR	After CR	P
LVEF (%)	30.0 ± 7.5	30.9 ± 7.7	0.067
NYHA class	2.38 ± 0.49	2.06 ± 0.51	0.00018
6-MWT distance (m)	417.8 ± 103.6	467.6 ± 98.4	0.016
Peak VO_2_ (mL × kg^−1^ × min^−1^)	18.7 ± 4.4	20.8 ± 4.7	0.025
Peak VO_2_% N	63.0 ± 15.4	71.1 ± 16.3	0.011
TFC (kOhm^−1^)	27.3 ± 5.2	25.6 ± 3.8	0.072

CR: cardiac rehabilitation, LVEF: left ventricular ejection fraction, ns: nonsignificant, NYHA: New York Heart Association, peak VO_2_: peak oxygen consumption, peak VO_2_% N: peak oxygen consumption as percentage of the normal value, SD: standard deviation, TFC: thoracic fluid content.

**Table 3:** Comparison of analysed parameters in subgroups distinguished by TFC.

	Low TFC *n* = 25	High TFC *n* = 25	*P*
Men, *n* (%)	22 (88.0)	22 (88.0)	—
Age (years), mean ± SD	57.8 ± 7.1	55.2 ± 9.9	0.305
BMI (kg × m^−2^), mean ± SD	30.2 ± 3.7	27.3 ± 3.4	0.006
Ischaemic aetiology of HF, *n* (%)	20 (80.0)	22 (88.0)	0.440
Myocardial infarction, *n* (%)	17 (68.0)	21 (84.0)	0.185
Diabetes, *n* (%)	8 (32.0)	6 (24.0)	0.529
Dyslipidaemia, *n* (%)	23 (92.0)	16 (64.0)	0.017
Hypertension, *n* (%)	14 (56.0)	12 (48.0)	0.571
ACE inhibitor, *n* (%)	22 (88.0)	24 (96.0)	0.297
Angiotensin receptor blocker, *n* (%)	5 (20.0)	1 (4.0)	0.082
Beta-blocker, *n* (%)	25 (100.0)	25 (100.0)	—
Loop diuretic, *n* (%)	23 (92.0)	17 (68.0)	0.034
Spironolactone, *n* (%)	24 (96.0)	23 (92.0)	0.551
Aspirin, *n* (%)	21 (84.0)	22 (88.0)	0.684
Statin, *n* (%)	23 (92.0)	23 (92.0)	—
NYHA class before CR, mean ± SD	2.40 ± 0.50	2.36 ± 0.50	0.776
NYHA class after CR, mean ± SD	2.00 ± 0.50	2.12 ± 0.52	0.412
LVEF before CR (%), mean ± SD	30.2 ± 6.8	29.8 ± 8.4	0.867
LVEF after CR (%), mean ± SD	31.6 ± 6.8	30.3 ± 8.0	0.551
LVEF change after CR (%), mean ± SD	1.44 ± 3.54	0.48 ± 2.20	0.255
6-MWT distance before CR (m), mean ± SD	400.0 ± 107.4	435.7 ± 98.8	0.226
6-MWT distance after CR (m), mean ± SD	454.4 ± 98.5	490.8 ± 98.5	0.347
6-MWT distance change after CR (m), mean ± SD	54.4 ± 64.7	55.1 ± 59.6	0.600
Peak VO_2_ before CR (mL × kg^−1^ × min^−1^), mean ± SD	18.9 ± 5.1	18.3 ± 3.6	0.686
Peak VO_2_ after CR (mL × kg^−1^ × min^−1^), mean ± SD	20.2 ± 4.6	21.4 ± 4.9	0.468
Peak VO_2_ change after CR (mL × kg^−1^ × min^−1^), mean ± SD	1.3 ± 2.3	3.1 ± 2.5	0.011
Peak VO_2_% N before CR, mean ± SD	66.8 ± 16.5	60.7 ± 13.9	0.113
Peak VO_2_% N after CR, mean ± SD	71.7 ± 15.6	70.7 ± 17.1	0.546
Peak VO_2_% N change after CR, mean ± SD	4.9 ± 7.9	10.1 ± 8.1	0.028
TFC before CR (kOhm^−1^), mean ± SD	23.0 ± 2.5	31.6 ± 3.2	<0.00001
TFC after CR (kOhm^−1^), mean ± SD	23.6 ± 3.0	27.6 ± 3.6	0.0001
TFC change after CR (kOhm^−1^), mean ± SD	0.7 ± 3.0	**−**4.0 ± 3.5	<0.00001

ACE: angiotensin-converting enzyme, BMI: body mass index, CR: cardiac rehabilitation, LVEF: left ventricular ejection fraction, ns: nonsignificant, NYHA: New York Heart Association, peak VO_2_: peak oxygen consumption, peak VO_2_% N: peak oxygen consumption as percentage of the normal value, TFC: thoracic fluid content.

**Table 4:** Comparison of analysed parameters in subgroups distinguished by peak VO_2_ improvement.

	Peak VO_2_ improvement *n* = 32	Peak VO_2_ no improvement *n* = 18	*P*
Men, *n* (%)	29 (90.6)	15 (83.3)	0.446
Age (years), mean ± SD	55.6 ± 9.0	58.2 ± 7.7	0.308
BMI (kg × m^−2^), mean ± SD	28.1 ± 3.5	29.9 ± 4.1	0.101
Ischaemic aetiology of HF, *n* (%)	26 (81.3)	16 (88.9)	0.479
Myocardial infarction, *n* (%)	25 (78.1)	13 (72.2)	0.639
Diabetes, *n* (%)	7 (21.9)	7 (38.9)	0.198
Dyslipidaemia, *n* (%)	24 (75.0)	15 (83.3)	0.495
Hypertension, *n* (%)	14 (43.8)	12 (66.7)	0.120
ACE inhibitor, *n* (%)	28 (87.5)	18 (100.0)	0.118
Angiotensin receptor blocker, *n* (%)	4 (12.5)	2 (11.1)	0.884
Beta-blocker, *n* (%)	32 (100.0)	32 (100.0)	—
Loop diuretic, *n* (%)	23 (71.9)	17 (94.4)	0.055
Spironolactone, *n* (%)	31 (96.9)	16 (89.9)	0.254
Aspirin, *n* (%)	28 (87.5)	15 (83.3)	0.684
Statin, *n* (%)	29 (90.6)	17 (94.4)	0.632
NYHA class before CR, mean ± SD	2.31 ± 0.47	2.50 ± 0.51	0.200
NYHA class after CR, mean ± SD	2.00 ± 0.44	2.17 ± 0.62	0.273
LVEF before CR (%), mean ± SD	30.6 ± 7.4	28.8 ± 7.9	0.426
LVEF after CR (%), mean ± SD	32.0 ± 7.5	29.0 ± 7.9	0.187
LVEF change after CR (%), mean ± SD	1.4 ± 3.4	0.2 ± 1.8	0.220
6-MWT distance before CR (m), mean ± SD	417.9 ± 103.5	417.7 ± 107.1	0.993
6-MWT distance after CR (m), mean ± SD	481.5 ± 90.1	442.8 ± 110.0	0.184
6-MWT change after CR (m), mean ± SD	63.6 ± 69.8	33.6 ± 47.3	0.054
TFC before CR (kOhm^−1^), mean ± SD	28.4 ± 5.5	25.3 ± 4.0	0.039
TFC after CR (kOhm^−1^), mean ± SD	25.7 ± 4.1	25.5 ± 3.4	0.850
TFC change after CR (kOhm^−1^), mean ± SD	2.7 ± 3.6	0.2 ± 4.1	0.012

ACE: angiotensin-converting enzyme, BMI: body mass index, CR: cardiac rehabilitation, LVEF: left ventricular ejection fraction, ns: nonsignificant, NYHA: New York Heart Association, peak VO_2_: peak oxygen consumption, TFC: thoracic fluid content.
